# ADC values compared to tumor grade and Ki-67 proliferation index detected by a digital image analysis program in meningiomas

**DOI:** 10.1177/02841851251365512

**Published:** 2025-08-17

**Authors:** Hanife Ersay, Hatice Gul Hatipoglu, Servet Guresci

**Affiliations:** 1 Department of Radiology, 12369 Ankara Bilkent City Hospital, Ankara, Türkiye; 2 Department of Radiology, Faculty of Medicine, Laval University, Quebec City, Canada; 3 Department of Pathology, 536164 Ankara Bilkent City Hospital, Ankara, Türkiye

**Keywords:** Meningioma, apparent diffusion coefficient, grade, Ki-67 proliferation index, digital image analysis

## Abstract

**Background:**

Meningiomas are the most common extra-axial tumors of the central nervous system, and accurate preoperative assessment of their histological grade is essential for effective treatment planning.

**Purpose:**

To investigate the relationship between the apparent diffusion coefficient (ADC) sequence, histopathological grade, and Ki-67 proliferation index for radiologically identifying meningiomas with poor prognosis.

**Material and Methods:**

The study included 90 patients with histopathologically confirmed meningioma between March 2019 and February 2021. The Ki-67 proliferation index was assessed using an image analysis program. Retrospectively, ADC maps and diffusion-weighted imaging (DWI) were reviewed. An oval-shaped region of interest was placed over the lesion's solid component and the normal-appearing white matter in the opposite hemisphere. Each patient's ADC ratio (ADC meningioma/ADC normal-appearing white matter) was calculated. The relationship between ADC and Ki-67 proliferation index was investigated, and ADC values of benign and atypical meningiomas were compared. Independent sample *t*-test, Mann–Whitney U test, and receiver operating characteristic were used for statistical assessment.

**Results:**

The mean ADC value was 844.11 ± 123.55 mm^2^/s for low-grade and 743.75 ± 92.64 mm^2^/s for high-grade meningiomas. The mean ADC ratio was 1.11 ± 0.19 for low-grade and 1.00 ± 0.15 for high-grade meningiomas. Both ADC values and ADC ratio significantly distinguished histopathologic grades (*P* = 0.003, *P* = 0.030, respectively). No significant correlation was found between ADC values or ADC ratio and the Ki-67 proliferation index (r = −0.123, *P* = 0.248; r = 0.033, *P* = 0.755).

**Conclusion:**

A statistically significant difference was found between ADC values and ADC ratio of low- and high-grade meningiomas. There was no correlation between either ADC values or ADC ratio and Ki-67 proliferation index.

## Introduction

Meningiomas are the most common central nervous system tumors, accounting for 20%–25% of all primary intracranial neoplasms. They are frequently intradural and arise from clusters of arachnoid cap cells in the outer arachnoid membrane. Although the etiology is unknown, established risk factors include radiation exposure and genetic conditions, such as neurofibromatosis type 2, which may lead to multiple lesions. Most occur in the supratentorial region, especially the parasagittal area, cerebral convexity, parafalcine region, and intraventricular spaces. The sphenoid wing and olfactory groove are the most common skull base sites (1).

According to the World Health Organization (WHO) classification, most meningiomas are histopathologically grade 1 (G1) and benign, grow slowly, and are surgically resectable depending on the location (1). In a meningioma presenting with classical histomorphological characteristics, increased mitotic activity (≥4 mitoses in 10 high power fields) or the presence of brain invasion are major criteria that contribute to the progression of the tumor to grade 2 (G2). In addition, the presence of more than three criteria, such as increased cellularity, small cell change, pattern loss, prominent nucleoli, and spontaneous/geographic necrosis, is considered G2. These tumors have a higher risk of local recurrence. Lesions presenting with either malignant histology similar to sarcoma, carcinoma, malignant melanoma, or with high mitotic activity (≥20 mitoses in 10 high power fields) are classified as grade 3 (G3). These are fatal anaplastic tumors (2).

In addition to histopathologic grading, the Ki-67 proliferation index is another prognostic factor that reflects the biological behavior of meningiomas. Although not part of the grading criteria, it has been reported as significantly increased from benign to atypical and malignant forms of meningiomas. It is expressed in all cell cycle phases except G0. Therefore, Ki-67 immunostaining is widely used as a reliable proliferation marker in routine practice (3).

**Fig. 1.**
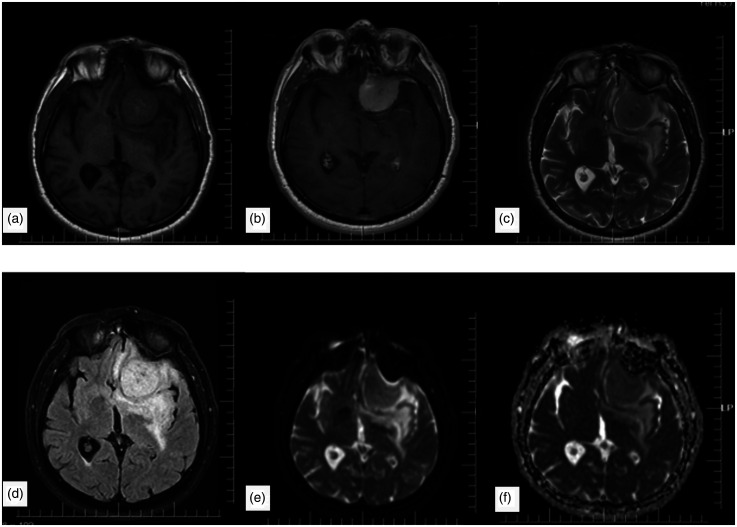
A meningioma case in the left frontal region. Hypo-isointense on (a) T1-weighted and (c) T2-weighted images, (b) intensely enhancing after contrast administration, and (d) hyperintense on FLAIR images. There is mild peripheral edema around the tumor. (e) DWI and (f) ADC maps show no significant diffusion restriction. ADC, apparent diffusion coefficient; DWI, diffusion-weighted imaging; FLAIR, fluid-attenuated inversion recovery.

Magnetic resonance imaging (MRI) is the most important imaging modality in the diagnosis and characterization of meningiomas. Typical meningiomas are isointense or hypointense on T1-weighted (T1W) images and isointense or hyperintense on both T2-weighted (T2W) images and fluid-attenuated inversion recovery (FLAIR) images. After contrast administration on T1W, meningiomas often show homogeneous, dense, and gradually increasing contrast enhancement (Fig. 1) (4).

DWI is an advanced MRI sequence that examines the microscopic movement of water molecules in different tissues, and ADC is a quantitative parameter derived from DWI. In some studies, ADC values were found to be associated with tumor grade and cellularity (5,6). Factors such as high nucleus/cytoplasm ratio, increased cellularity, small cell size, the presence of fibrotic and gliotic foci within the tumor, and tumor perfusion may be factors for low ADC values in high-grade tumors (6).

Although conventional MRI is valuable in diagnosing meningiomas, its ability to determine tumor grade is limited. Accurate preoperative grading is essential for guiding treatment, as low-grade meningiomas may be managed with observation or surgery alone, while high-grade tumors often require adjuvant therapies (7). DWI and ADC have emerged as promising non-invasive tools for assessing tumor aggressiveness by reflecting cellularity. Recent studies and meta-analyses suggest that ADC values may help distinguish tumor grades and correlate with proliferation markers such as the Ki-67 proliferation index (8). The aim of the present study was to investigate the association between ADC values, tumor grade, and Ki-67 proliferation index to support preoperative assessment and radiology-based treatment planning.

## Material and Methods

Approval was obtained from the University of Health Sciences Non-Interventional Clinical Research Ethics Committee (approval no: E1-21-1777 dated 28 April 2021). The requirement for informed consent was waived due to the retrospective nature of the study.

### Patient selection

The study included 90 patients (age range = 33–78 years) who were histopathologically diagnosed with meningioma after surgery between March 2019 and February 2021 at Ankara Bilkent City Hospital. Preoperative images lacking DWI and ADC maps or with severe artifacts were excluded from the study. In addition, patients who had previously undergone surgery and radiotherapy for their lesions, as well as those with meningiomas featuring small solid components that were difficult to measure, were excluded. In cases with multiple lesions, only the lesion that was histopathologically confirmed as meningioma after surgery was enrolled.

### Radiological evaluation

The study was performed with General Electric Signa Pioneer with 3T power. The MRI protocols were as follows: axial T1W images: echo time (TE)/repetition time (TR) = 9.24/2363 ms, field of view (FOV) = 24 × 18 cm, matrix = 360 × 260; axial FLAIR images: TE/TR = 97.5/8200 ms, FOV = 24 × 18 cm, matrix = 320 × 239, section thickness = 5 mm; axial T2W images: TE/TR = 113/4984 ms, FOV = 24 × 18 cm, matrix = 320 × 320, section thickness = 5 mm; and postcontrast axial T1W images: TE/TR = 8/675 ms, FOV = 24 × 18 cm, matrix = 256 × 256, section thickness = 5 mm, and slice gap = 1 mm. For DWI in the axial plane, the imaging protocol performed at TE/TR = 86/3750 ms, FOV = 24 × 18 cm, matrix = 128 × 128, section thickness = 5 mm, along with diffusion-sensitive gradients applied with two different b-values (b = 0, b = 1000) in each of the three directions (x, y, z). ADC maps were automatically generated by workstations.

Conventional MRI and DWI were evaluated together, and the region of interest (ROI) was placed on ADC maps to correspond to the solid component of the lesion, avoiding cystic-necrotic, calcified, and hemorrhagic areas of the lesion by evaluating T1W, T2W, and contrast-enhanced T1W images in conventional MRI. ADC values were then calculated. In addition, control ADC measurements were taken from normal-appearing white matter in the opposite hemisphere. ADC ratio (ADC meningioma/ADC normal-appearing white matter) was calculated for each patient. The measurements were taken in an oval-shaped ROI with a range of 0.8–1.1 cm^2^.

### Histopathologic evaluation

**Fig. 2.**
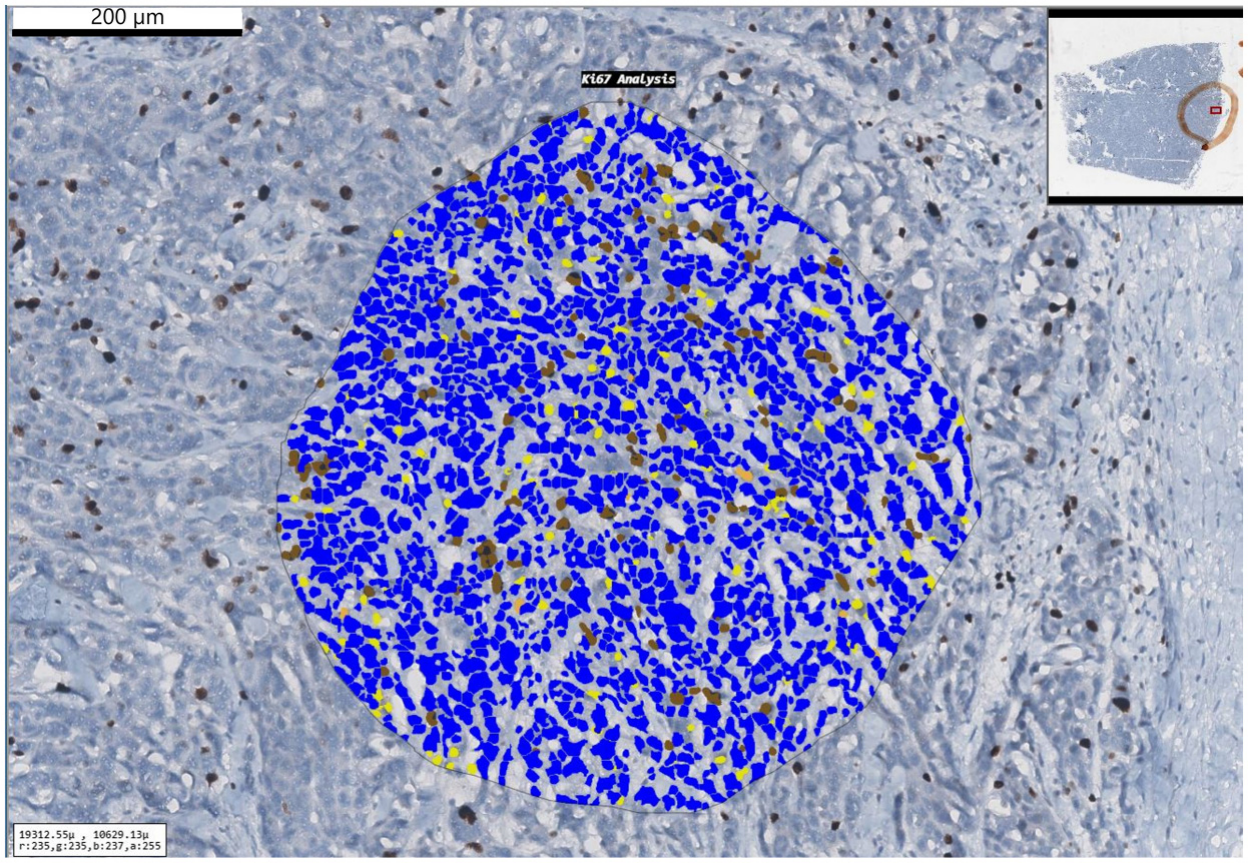
A case of atypical meningioma with a Ki-67 proliferation index of 12%.

All the biopsy samples were fixed in formalin and processed in automated machine (Tissue-Tek VIP 6 AI; Sakura Finetek, Tokyo, Japan). The tissues were embedded in paraffin blocks and standard 4-µm thick slices were prepared. The slides were stained with hematoxylin and eosin (H&E). An experienced neuropathologist (SG) reviewed the slides according to WHO 2021 criteria under the light microscope (Eclipse Ni-U; Nikon Corporation, Tokyo, Japan). The sections best reflected the tumor were immunohistochemically (IHC) stained with Ki-67 primary antibody (clone 30-9, Ventana, 1/100 dilution, 20 min). Slides were scanned at 0.25 µm/pixel resolution with a Aperia CS2 digital scanner (Leica, Deer Park, USA). A digital image analysis program was used to quantitate Ki-67 proliferation index (Virapath-2; Virasoft Software Inc., Istanbul, Türkiye). Hot-spot areas were hand-annotated under pathologist supervision. In total, 1000 and 500 cells were counted and the results were stated in percentages (Fig. 2).

### Statistical analysis

The data analysis was performed using SPSS version 22.0 (IBM Corp., Armonk, NY, USA) and Excel software (Microsoft Corp., Redmond, WA, USA). For statistical comparison, G1 tumors and G2 tumors were grouped. To ensure that statistical analyses were conducted with the correct tests, the analyses included histogram drawing, evaluation of central and prevalence criteria, and conformity to normal distribution (Kolmogorov–Smirnov test). For detecting differences, the *t*-test, Mann–Whitney U-test, and ROC analysis for optimal threshold values were used. The results were evaluated at a 95% confidence interval (CI) and *P* <0.05 indicated statistical significance.

## Results

Of the 90 patients with meningioma included in the study, 54 (60%) were women and 36 (40%) were men. The numbers of patients allocated to G1 and G2 were 74 and 16, respectively.

The mean ADC value was 844.11 ± 123.55 mm^2^/s (range = 607.6–1240 mm^2^/s) for G1 lesions and 743.75 ± 92.64 mm^2^/s (range = 575–857 mm^2^/s) for G2 lesions, and the difference was statistically significant (*P* = 0.003). According to the ROC curve analysis, ADC was found to be a statistically significant marker for the differentiation between G1 and G2 (area under the ROC curve [AUC] = 0.738, 95% CI = 0635–0825; *P* = 0,0001).

The ADC ratio was calculated by dividing the ADC values measured from the lesions by the ADC values measured from the contralateral white matter. The mean ADC ratio was 1.09 ± 0.18 (range = 0.73–1.82). The mean ADC ratio was 1.11 ± 0.19 (range = 0.77–1.82) for G1 and 1.00 ± 0.15 (range = 0.73–1.25) for G2 (Table 1).

**Table 1.** Distribution of the ADC ratio by histopathologic grade.

	G1	G2
ADC ratio	1.11 ± 0.19 (0.77–1.82)	1.00 ± 0.15 (0.73–1.25)

Values are given as mean ± SD (range).

ADC, apparent diffusion coefficient.

**Fig. 3.**
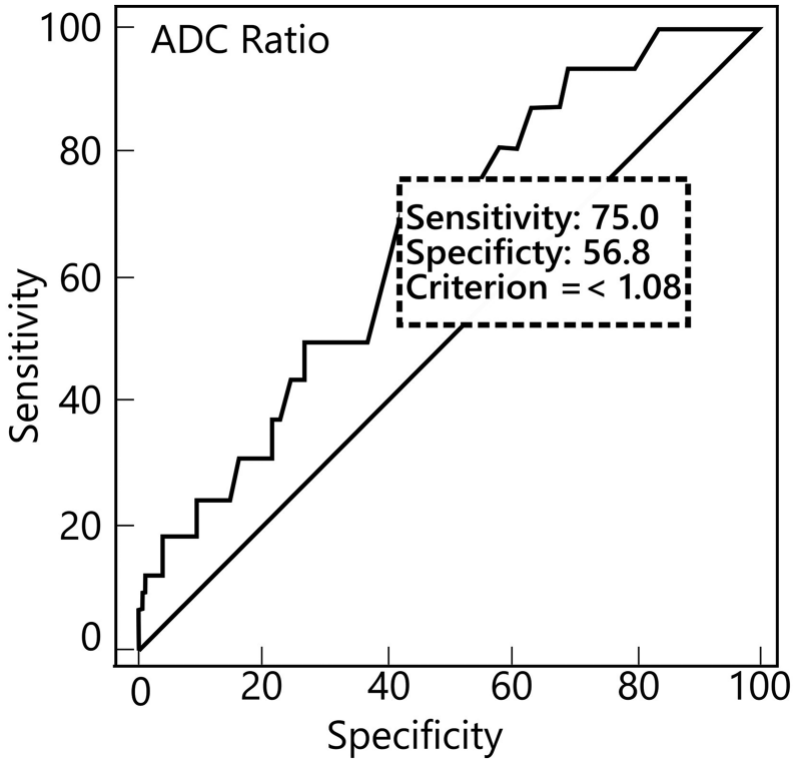
ROC distribution of the ADC ratio by histopathologic grade. ADC, apparent diffusion coefficient; ROC, receiver operating characteristic.

According to the ROC analysis, the ADC ratio was found to be a statistically significant marker for the differentiation between G1 and G2 (AUC = 0.669, 95% CI = 0562–0765; *P* = 0,0135) (Fig. 3).

The mean Ki-67 proliferation index distribution of the lesions was 5.30 ± 4.74 (range = 0.5–24). As for the mean distribution of Ki-67 proliferation indices by histopathologic grade, it was 4.48 ± 3.61 (range = 0.7–16.87) for G1 and 9.08 ± 7.17 (range = 0.5–24) for G2, and the difference was statistically significant (*P* = 0.004) (Table 2).

**Table 2.** Distribution of the Ki-67 proliferation index by histopathologic grade.

	Grade 1	Grade 2
Ki-67 proliferation index	4.48 ± 3.61 (0.70–16.87)	9.08 ± 7.17 (0.50–24.00)

Values are given as mean ± SD (range).

**Fig. 4.**
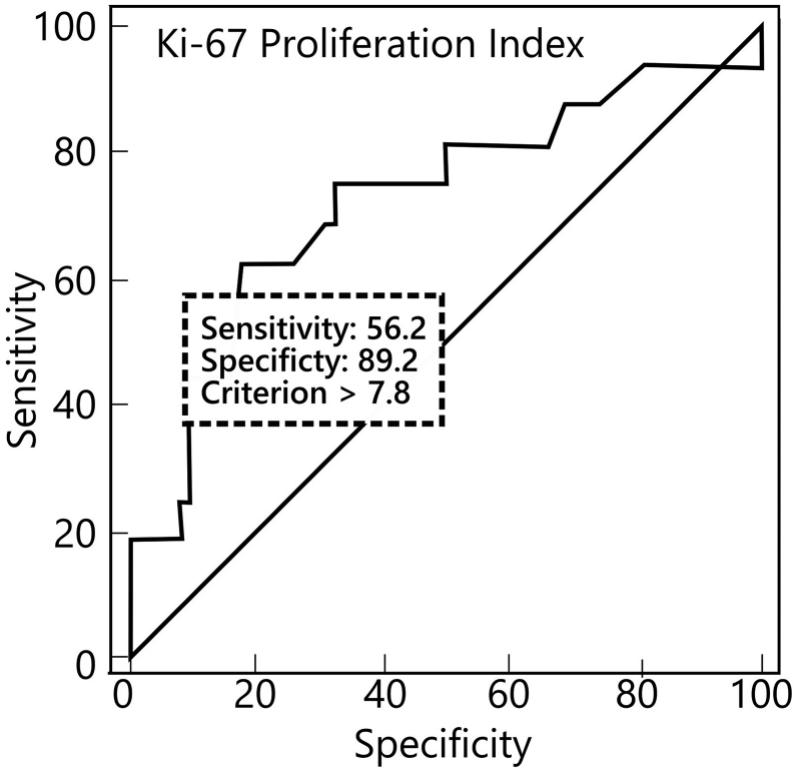
ROC distribution of the Ki-67 proliferation Index by histopathologic grade. ROC, receiver operating characteristic.

According to the ROC analysis, the Ki-67 proliferation index was found to be a statistically significant marker for the distinction between G1 and G2 (AUC = 0.73; 95% CI = 0.628–0.819; *P* = 0.0024). For a Ki-67 proliferation index threshold value of 7.80, sensitivity was 56% and specificity was 89% (Fig. 4).

**Fig. 5.**
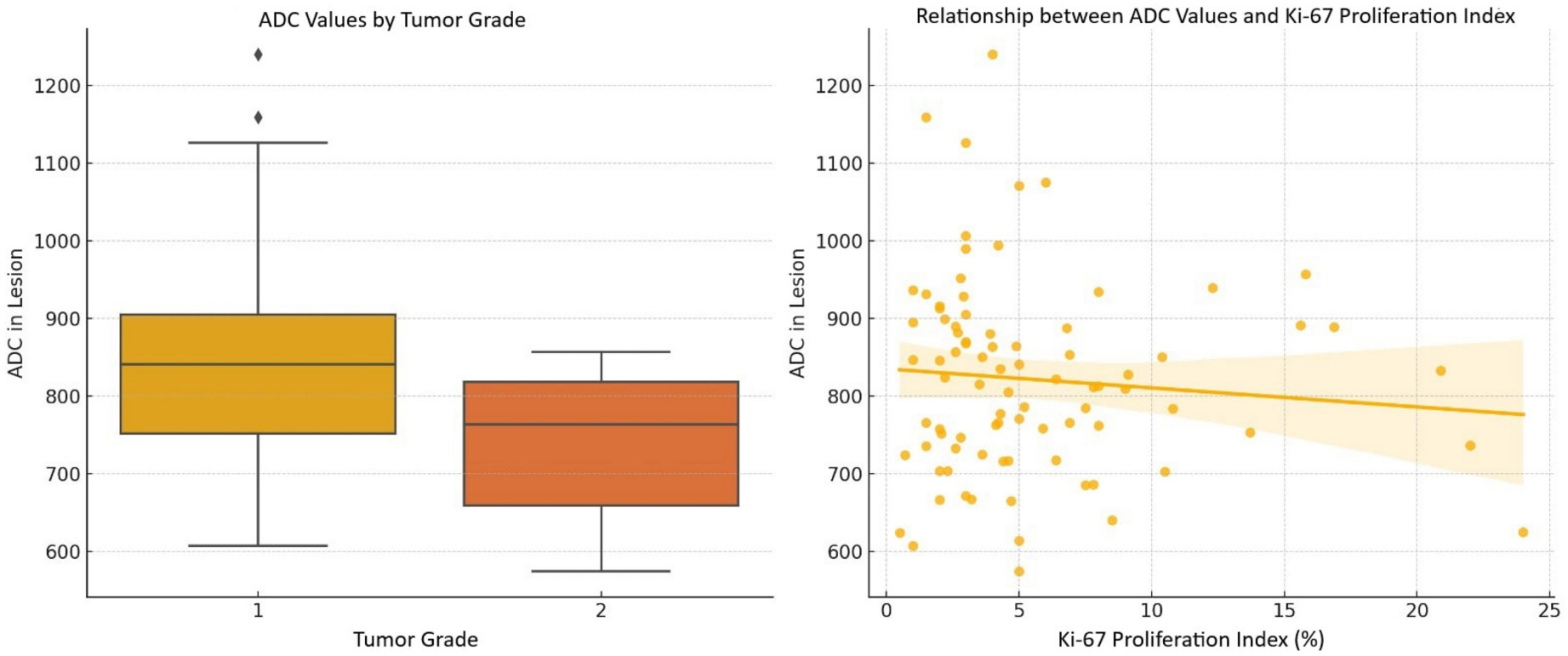
Comparison of ADC values across tumor grades and their correlation with Ki-67 proliferation index. Left: G1 tumors have higher median ADC values compared to G2 tumors. Right: a weak negative correlation between ADC values and Ki-67 proliferation index. ADC, apparent diffusion coefficient.

There was no statistically significant correlation between the ADC ratio and Ki-67 proliferation index (r = −0.074; *P* = 0.487). When G1 and G2 were analyzed separately, no statistically significant correlation was found between the ADC ratio and the Ki-67 proliferation index (r = −0.012, *P* = 0.917; r = −0.080, *P* = 0.767). In addition, a weak negative correlation was observed between ADC values and Ki-67 proliferation index, which was not statistically significant (r = −0.123; *P* = 0.248). When analyzed separately by histopathologic grade, no statistically significant correlation was found between ADC value and the Ki-67 proliferation index (r = 0.011, *P* = 0.925; r = −0.075, *P* = 0.782) (Fig. 5).

## Discussion

In the present study, in which we investigated the relationship between ADC values, the histopathologic grades of meningiomas, and their Ki-67 proliferation indices, we found a statistically significant correlation between ADC values and histopathological grade.

Some studies found that preoperative ADC values are reliable predictors for differentiating between tumor grades (6,9–13), while others indicated this parameter may not provide significant diagnostic value (14,15). Although an obvious overlap was identified, we found a statistically significant mean difference in ADC between low- and high-grade meningiomas (844.11 ± 123.55 mm^2^/s in G1 meningiomas and 743.75 ± 92.64 mm^2^/s in G2 meningiomas; *P* = 0.003). In a study by Bozdağ et al., in which all ADC values were significantly lower in high-grade meningiomas than in low-grade meningiomas (*P* <0.05), ROI measurements were performed on at least two consecutive slices of the lesions to determine the maximum, mean, and minimum ADC values. In addition, ROI measurements were taken from the contralateral centrum semiovale, and the ADC ratio (ADClesion/ADCwhite matter) was calculated for each patient (9). Similarly, Atalay et al. performed standard ROI measurements on three consecutive sections of the lesions to determine the range and mean ADC values, and calculated the ADC ratio using contralateral white matter. In their study of 45 patients, the ADCmin value was found to be statistically significant for differentiating tumor grade (*P* = 0.018) (10).

In a study of 177 cases conducted by Sanverdi et al. (14), there was no significant difference between the mean ADC values and ADC ratios of meningiomas of different histopathological grades (1.22 ± 0.07 for G1 tumors, 1.05 ± 0.1 for G2 tumors, and 0.96 ± 0.2 for G3 tumors). The ADC value was obtained as the mean of three measurements from the various parts of the lesion. In addition, DWI applied with three different b-values with a maximum of 1000 s/mm^2^. In another retrospective study that found no correlation between histopathologic grade and ADC ratio, mean ADC ratios were 1.266 ± 0.29 and 1.185 ± 0.115 for low- and high-grade meningiomas, respectively (*P* = 0.2). DWI was performed with two b-values of 0 and 800 s/mm^2^ (15).

In our study, we found a statistically significant correlation between mean ADC ratio and histopathologic grade (0.92 ± 0.15 in low-grade and 1.02 ± 0.16 in high-grade meningiomas; *P* = 0.003). In a retrospective study of 44 meningioma cases conducted by Baskan et al. (12), ADC ratio was calculated by comparing ADC of the lesions to ADC of normal white matter at the level of the centrum semiovale. In their study, mean ADC ratios were close to our cohort (0.91 ± 0.12 and 1.12 ± 0.18 in high- and low-grade meningiomas, respectively), and the difference was statistically significant (*P* = 0.002). They obtained six ROI measurements from three consecutive slices and calculated the mean ADC value. Both their study and ours used a 3-T MRI scanner and employed b-values of 0 and 1000 s/mm² for DWI and ADC calculation, indicating a high level of technical comparability between the imaging protocols. Although we acknowledge that multiple ROI measurements may yield more accurate results, the technical consistency between the two studies—along with the similarity of the findings—supports the validity of comparing results across both cohorts and strengthens the reliability of the ADC ratio as a potential imaging biomarker for meningioma grading.

Variations in ADC, ADC ratio, and histopathologic grade of meningiomas among studies could be linked to factors such as scanning features, the number of cases, different subtypes enrolled in the studies, and the methods used for ROI measurement and placement. In our study all 90 images were obtained with a 3-T power MRI. Studies with larger patient populations that did not find a correlation between ADC or ADC ratio and histopathologic grade were conducted using 1.5-T and 1-T MRI machines, respectively (14,15).

Some recent studies have examined how MRI field strength affects ADC measurements. A prospective study by Merhemic et al. comparing ADC values between 1.5-T and 3-T scanners in the same patients found no significant difference, demonstrating comparable ADC reproducibility under standardized conditions (16). A phantom study by Lavdas et al. reported minor ADC differences between 1.5-T and 3-T scanners but noted that 3-T imaging might be more affected by technical limitations, potentially influencing measurement quality (17). Therefore, the advantages of 3-T MRI in ADC measurement are not absolute and depend on multiple factors including scanner technology, acquisition protocols, and image processing methods. These technical considerations may partly explain the inconsistent findings across studies regarding ADC values and tumor grading.

We measured the ROI area from the solid part of the lesion, in the range of 0.8–1.1 cm^2^. Overestimating the ROI area could lead to measurements from cystic–necrotic areas. In addition, a small ROI area may not provide accurate information about the ADC value of the lesion. We also conducted a single measurement for each lesion. Some studies obtained multiple ROI measurements to calculate the mean ADC value (9,10,12,14), which could potentially yield more reliable insights into the lesion's structure and better capture tumor heterogeneity.

In the present study, we compared the ADC value and ADC ratio with the Ki-67 proliferation index and found no correlation between them. Similar results were reported in the literature (18,19). In a retrospective study comparing the Ki-67 proliferation index and perfusion MRI features in high-grade meningiomas, although the mean Ki-67 value in G2 meningiomas (14.5%, range = 2%–28%) was lower than in G3 meningiomas (19.0%, range = 3%–38%), the difference was not statistically significant (*P* = 0.61) (18). In a meta-analysis of 1055 meningioma cases, Meyer et al. observed a statistically weak correlation between ADC and the Ki-67 proliferation index, and a significant overlap of ADC values of histopathological grades (r = −0.36) (19). There are also studies that have demonstrated a relationship between ADC and Ki-67 proliferation index (9–13). Bozdağ et al. found significant negative correlations between ADC values and ADC ratio and Ki-67 proliferation index (*P* < 0.05 for all ADC and ADC ratio measurements) (9). In the study by Atalay et al., although a statistically significant inverse correlation was found between ADCmin and the Ki-67 proliferation index, no such significance was observed for ADCmean (10). In a study by Zhang et al., where the Ki-67 proliferation index was available only for eight patients, a statistically significant inverse correlation was found between the Ki-67 proliferation index and ADC ratio (*P* = 0.03) (20). In studies that found a relationship between ADC values and K-67 proliferation index, DWI was conducted using two b-values, 0 and 1000 s/mm^2^, which aligns with our study's methodology. In the study by Baskan et al., ROI measurements were taken within a range of 0.2–1 cm^2^, using a 3-T MRI scanner. Moreover, the mean ADC value was calculated through multiple ROI measurements (12). Using mean ADC values may provide more accurate information about the lesion compared to single ROI measurement. In a multicenter study with Ki-67 proliferation index measurements of 73 meningioma cases, Surov et al. found an inverse correlation between the Ki-67 proliferation index and ADC (r = −0.63, *P* <0.001). In the study ROI measurements were drawn around the margin of the lesion (13). However, performing a whole lesion measurement does not seem to provide the accurate ADC value of the lesion as it may contain non-cellular areas. Furthermore, when considering the number of patients, the present study included more patients compared to those studies that established a correlation between ADC and Ki-67 proliferation index.

Although the primary focus of this study is radiological evaluation, the Ki-67 proliferation index remains an important pathological marker in meningioma grading. According to our ROC analysis, a threshold of 7.8% provided a reasonable balance between sensitivity and specificity for differentiating G1 and G2 tumors. Although the sensitivity at this cutoff is moderate (56%), the high specificity (89%) may help reduce false-positive results in clinical settings. Further sensitivity analyses with alternative thresholds (e.g. 5% or 10%) showed trade-offs between sensitivity and specificity, suggesting that the chosen cutoff provides a balanced and practical option for this cohort.

The Ki-67 proliferation index is generally analyzed semi-quantitatively in routine practice and is prone to variation among observers. To overcome this dilemma, we employed an image analysis program to calculate the index with greater sensitivity. Therefore, we believe that our data are more objective. To the best of our knowledge, this is the first study in which the Ki-67 proliferation index analyzed by a digital image analysis program is compared with ADC values of meningiomas.

Regardless of the technique used in the studies, the primary reason for conflicting results on the correlation between ADC values and the Ki-67 proliferation index could be the differing microstructures among meningioma subtypes, leading to various ADC values. The differences in ADC values may also arise from not only cellularity but also the presence of various intracellular proteins, which can influence the movement of intracellular water and consequently impact ADC values (6). Future studies with larger cohorts should consider performing subtype-specific analyses to better understand the heterogeneity in ADC values and Ki-67 proliferation index correlations among different meningioma subtypes. Moreover, because the ROI placement for ADC measurement may not correspond to the area where the histopathological sampling was performed, the results could vary.

Another method to assess the aggressiveness of meningiomas is positron emission tomography (PET), a functional imaging modality. A recent study found that compared to MRI, PET, especially when using somatostatin receptor tracer ^68^Gallium-DOTATATE, provides additional information to the diagnosis of meningiomas (21). Therefore, PET may potentially reveal the histopathologic features of meningiomas better. In addition to current neuroimaging techniques, artificial intelligence (AI) models have recently been employed to enhance the understanding of meningiomas structure and to differentiate between low- and high grade meningiomas. Even if it has not yet been fully integrated into clinical practice, its potential for diagnosing, grading, and characterizing meningiomas biological behaviors is apparent (22,23).

The observed difference in mean ADC values between low- and high-grade meningiomas in our study (844.11 ± 123.55×10^−3^ mm²/s vs. 743.75 ± 92.64 ×10^−3^ mm²/s, respectively; *P* = 0.003) suggests that diffusion characteristics could be incorporated into preoperative clinical decision-making. An ADC value at approximately 750 × 10^−3^ mm²/s or below may raise suspicion for atypical histopathology, even if conventional imaging and intraoperative appearance suggest a benign tumor. In such cases, earlier consideration of adjuvant radiotherapy or more intensive follow-up imaging may be warranted. For example, surveillance protocols might include shorter imaging intervals for G1 meningiomas with lower ADC values, particularly if complete resection was not achieved. Although prospective studies are needed for validation, our findings highlight the potential of ADC as a non-invasive tool to guide clinical management.

The present study has some limitations. First, the retrospective design of the study and the small number of patients represent important limitations that may affect the generalizability and validity of our findings. Moreover, retrospective design may have contributed to the spatial mismatch between ADC measurements and histopathological sampling sites. Future studies could reduce this limitation by using image-guided biopsy or neuronavigation systems to better match ROIs with histopathology sampling sites (24). Improving the alignment between radiology and pathology data could also improve the accuracy of the correlation between ADC values and both tumor grade and the Ki-67 proliferation index. In addition, prospective studies where radiologists and pathologists decide together on the sampling areas may help achieve better agreement between imaging and tissue analysis and reduce spatial mismatch. In addition, the absence of G3 meningioma cases, mainly due to its rarity, is a significant limitation of our study. This limits the generalizability of our results across the full pathological spectrum of meningiomas. Collaborative multicenter studies could address this by increasing the representation of rare high-grade tumors. In addition, stratifying analyses according to WHO grade prevalence may further enhance the applicability of imaging biomarkers across all tumor grades.

Meningiomas are generally more cellular and have a denser extracellular matrix than normal white matter. We used the ADC ratio (ADC meningioma/ADC normal-appearing white matter) to normalize diffusion values using white matter as a stable internal reference. We also believe that employing ratios has helped address and minimize measurement-related inaccuracies.

The primary aim of this study was to evaluate the relationship between ADC values, tumor grade, and the Ki-67 proliferation index in meningiomas. Although survival analysis was beyond the scope of our current dataset, incorporating long-term clinical outcomes in future studies could help clarify whether ADC value also has prognostic value. Such data may further support the use of ADC value in presurgical planning and risk stratification.

In conclusion, a statistically significant difference was observed in ADC values and ADC ratio between low- and high-grade meningiomas. In addition, no correlation was found between the Ki-67 proliferation index and ADC values or ADC ratio.
